# Digitization of Society: Alternative Projections of the Future

**DOI:** 10.1134/S1019331622120115

**Published:** 2022-09-29

**Authors:** N. M. Travkina

**Affiliations:** Institute for US and Canadian Studies, Russian Academy of Sciences, 121069 Moscow, Russia

**Keywords:** digitization, digital security, social spheres, scientific and technological formation, digital project, coronavirus pandemic, digital divide, digital utopias and dystopias

## Abstract

The impact of digitization on four spheres of society, i.e., economic, political, social, and spiritual, is analyzed. Digitization is defined as the diffusion of information and communication technologies (ICT) that can bring about both positive (legitimate) and negative (wrongful) effects. At the same time, today the digitization of public spheres directly involves the component of ensuring the digital security of society, which is becoming increasingly global in character. Cyber wars and cyber attacks cause economic damage on a global scale, amounting to six trillion dollars US annually, which is commensurate with the economic losses of wartime. Large-scale digitization of public spheres for the first time in human history creates an objective opportunity for constructing and designing future social states, which makes a fundamental difference between the course of future socio-economic and political processes and the historical evolution of the previous eras. This gave rise to a dichotomy of virtual utopias and dystopias of Future projects. Dystopias are inspired by visions of the coming “digital slavery,” while utopias focus on visions of a “digital paradise.” Polarized views on the digital Future are based on the processes of the “digital divide,” the meaning of which is that digitization contributes to a significant increase in inequality in access to digital goods, which in turn results in a growing inequality in the distribution of income and wealth. The coronavirus pandemic promoted a powerful acceleration of digitization processes, which acted as a form of society’s adaptation to its stresses and harmful consequences. Digitization has made social distancing possible and cost-effective. At the same time, the pandemic was conducive to a colossal increase in the economic power and political influence of digital corporations, which objectively requires a sharp increase in the regulatory role of the state, which should put digitization under effective public control.

## INTRODUCTION

### Global Digital Security

Digitization, the “brand identity” and the basis of which is the World Wide Web of the Internet, has become the most important material factor in accelerating global processes, determining their specificity and direction. As in previous eras, globalization turned out to be a two-faced Janus, on the one hand, bringing together socio-economic and political systems, and on the other, sowing seeds of destruction, discord, and wars between countries. Digitization, as a powerful force for global socio-economic and political transformations, can act as a potent tool for both creative and destructive processes. In its last capacity, it has the potential to turn, and perhaps has already turned, into a new type of collision—cyberwars, the destructive potential of which, in terms of economic losses, is comparable to the economic damage of the world wars in the first half of the twentieth century and financial and economic crises of the twenty-first century.

Digitization has brought with it, as a mandatory and integral element, the parameter of security; the digital Future must be secure, otherwise, there may be no chance for it at all. In this regard, we can mention the following fact: attacks in cyberspace aimed at obtaining economic benefits are already causing damage to the global economy in the range of $100 billion to $6 trln, and every year these losses increase [1 : 1].

## DIGITAL DIFFUSION IN SOCIETY

**Fig. 1.**
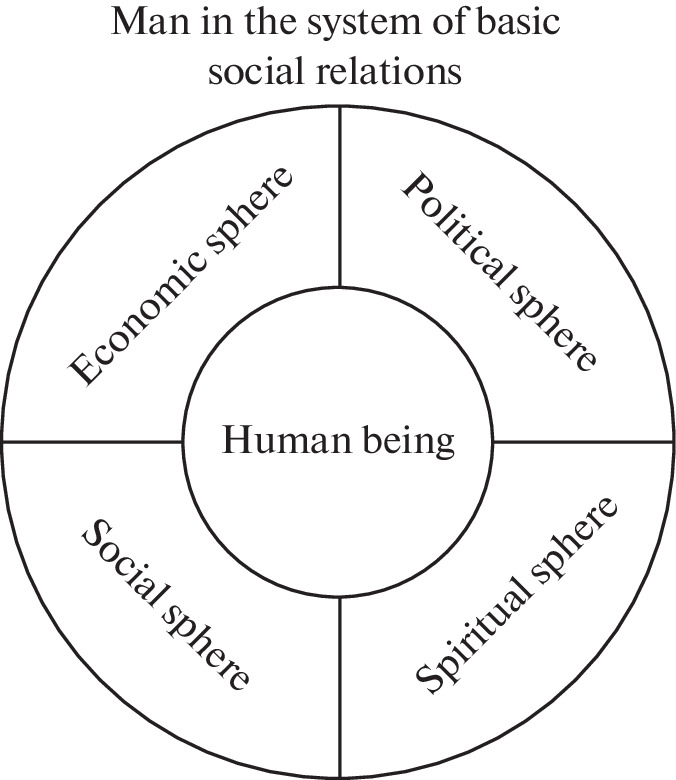
Human being in the system of basic social relations. * Spheres of life in society, Available at: https://www.grandars.ru/college/sociologiya/sfera-obshchestva.html

The canonical definition of digitization states that it represents the process of introducing digital technologies into various public spheres. The generally accepted classification of public spheres includes four realms, i.e., (1) economic, (2) political, (3) social, and (4) spiritual. Humans stand in the center of this classification matrix because the listed spheres reflect their basic social needs as individuals and personalities: the need to work and increase the material goods and services at one’s disposal; the need to participate in public and state life, reflecting one’s political preferences and views; the need for a social life, which stems primarily from belonging to a family, people, and various age and sex groups; and, finally, the need for spiritual development, which involves the development of worldview ideas and morality, and an improvement in the educational level. This “weaving” of an Individual into public relations in the broadest sense of the word is reflected in Fig. 1.

Historically, digital technologies appeared immediately after World War II, in the second half of the 1940s, but the modern history of information and communication technologies (ICT) begins in 1969, when the US Department of Defense put into operation the Arpanet computer network, which became the prototype of the modern Internet [2].

**Fig. 2.**
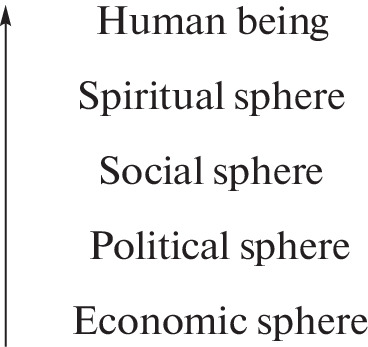
Gradual digitization of public spheres and man.

The diffusion of subsequent digital innovations was ascending, starting from the economic sphere, and gradually spreading to all other public spheres, and since the beginning of the twenty-first century, it has begun increasingly to involve man himself (concepts and practical application of artificial intelligence, AI). This incremental advance of digital technologies across the main public areas is schematically shown in Fig. 2.

The progressive digitization of public spheres, including that of an individual himself, makes us wonder about the legitimacy of digitizing each of those, which will ultimately enhance its functional efficiency in meeting human needs, and about the extent to which the intrusion of digital technologies into areas that occupy a higher place in their hierarchical order is a factor in the growing dysfunctionality of this area.

At present, it can be stated that the widespread use of digital technologies in the economy seems justified, since it reflects the logic of a progressive change in scientific and technical paradigms. The economic sphere is a legitimate area for the application and dissemination of digital technologies, although, as the Dutch sociologist M. Ossewaarde pointed out, “digital transformation can be interpreted as an assertion of the dominance of economic forces personified by the oligarchic power of technological clusters, the most famous of which is Silicon Valley” [Ossewaarde, p. 25]. With regard to other public spheres, it can be hypothetically assumed that their digitization looks more problematic and is accompanied by a growing mutual substitution of digital technologies for the qualities and properties of a Human as a sociobiological species, which includes, among other things, mental, sensual, and volitional areas.

It should be pointed out, for example, that the digitization of the political sphere can lead to a sharp tightening of political control over society and its manipulation in the interests of the “governing elites.” According to the German professor of political science J. Hofmann, authoritarian regimes “either cannot or do not want to rely on the willingness of their citizens to cooperate. Established democracies have many opportunities for the political mobilization of citizens: they can rely on the public to form a critical consensus, understanding, and solidarity. In addition, they can expect a community action from a significant part of their population. Instead of strengthening these aspects of political life, digital monitoring can only undermine them” [3].

The digitization of the social sphere can result in the weakening and even disintegration of social communities, including the institution of the family, and even entire societies. This danger, in particular, is pointed out by Norwegian sociologists professors H. Spilker and L. Reutter, who analyzed the problem of creating large databases for ordinary citizens by government bodies: “The use of large databases raises serious questions about privacy, data security, and ethics. When using artificial intelligence, these issues are, of course, even more critical in the public sector than in the private sector. At the same time, there is a significant potential for unauthorized control over citizens, as well as the risk of automation of unfair actions” [Reutter and Spilker, p. 96].

The digitization of the spiritual sphere in its finished form in the future means the complete leveling of values that are purely human, related to moral, ethical, and religious values. Digitization, in fact, cuts off all connections of a person with the spiritual dimension, because, as noted by the American theologian and engineer L. Dovich, “life in a community of people and constant communication with them is the currency of spiritual growth” [Dovich, p. 12].

Moreover, finally, the digitization of a person himself can be considered tantamount to the “self-destruction” of a person as *homo sapience,* a wise man, as modern biological science and every human being knows him. According to the popular historian Y. Harari, breakthroughs in biotechnology and information technology “will give us power over the inner world and allow us to change ourselves, but we do not understand the complexity of our mind, and these changes can have a devastating effect on our entire system of thinking” [Harari, p. 16].

## DIGITIZATION AND CONSTRUCTING VIRTUAL PROJECTIONS OF THE FUTURE

The traditional view of the course and evolution of historical processes boiled down to the fact that the temporal flow from the Past to the Future through the Present is autonomous, to a certain extent independent of the will and consciousness of individual social groups and societies as a whole. It is the ideas about the autonomous nature of the action performed by forces that determine the transition from one social formation to another, from one scientific and technological paradigm to another, that underlie the laws of the historical evolution of countries and humanity as a whole. The change in scientific and technological paradigms, starting from the end of the eighteenth century and ending with our time, is reflected in the Table 1.

**Table 1.** Scientific and technical paradigms, 1785–2022

Mechanization Water energy Iron	Steam engines Railways	Electricity Internal combustion engines Chemicals	Electronics Aerospace engineering	Internet Computers Biotechnology	Digitization
1st wave	2nd wave	3rd wave	4th wave	5th wave	6th wave
1785 *	1845 *	1900 *	1950 *	1990 *	2020 *
60 years**	55 years**	50 years**	40 years**	30 years**	25 years**

* Beginning of period.

** Length of period.

It should be noted that modern social sciences in their understanding of the general direction of the social, scientific, and technological evolution of humanity have not gone far from the statement of the founder of political economy A. Smith, who believed that the course of historical evolution is determined by the “invisible hand” of Providence.^1^ At the end of the twentieth century, a discussion began and continues in the foreign literature about the extent to which the references to the “invisible hand” in the three works of A. Smith can be considered nothing more than a metaphor, and to what extent they are a claim to a theoretical generalization, which was formulated by the great thinker of the end of the eighteenth century, but could not bring it to its logical conclusion. According to some researchers, the “invisible hand” can be considered the basis for far-reaching theoretical generalizations, especially when the question arises about the effectiveness of a “sound” state policy that leads to unforeseen consequences often opposite to the original intentions. This situation arises because public policy makers “carry out a course aimed at correcting certain problems, ignoring the opinion of critics who warn that the chosen direction of policy will lead to the opposite result, and then the responsibility for unforeseen results will be shifted to a third party to take the blame. As a result, unforeseen results are caused by elites who believe they have the knowledge and wisdom necessary to realize a “better society for all” [4, p. 9].

The digitization of social relations drastically changes the direction of their evolution, in which the Future can be designed in the Present. Social forces that have fully mastered and control the process of digitization claim that the society of the Future is not the result of the action of autonomous forces, in some cases relying on the help and support of the “invisible hand” of Providence, but arose as a result of the implementing the ***digital Project*** created in computers, supercomputers, and with the help of artificial intelligence (AI).

The most important factor that makes it theoretically possible to implement a digital project is the widespread use of digital technologies in all spheres of society. The wide diffusion of digital technologies leads to a historically unprecedented symbiosis of almost every person and computer bringing about an information society, which is “the result of the transition from the previous digital era to a new post-digital world in which digital has become the basis of everyday life” [Dufva and Dufva, March 2019, p. 18].

The basis of a possible digital project was the concept of a digital code (or algorithm) that can be “changed, updated, fixed, hacked, stored, and analyzed without changing the physical machine itself” [Dufva and Dufva, March 2019, p. 17]. In a historical retrospective, the change in scientific and technological paradigms manifested itself in the form of a visible change in the symbols and products of the achievements of scientific and technical thought in the form of steam engines, railway locomotives, cars, and aircraft, and achievements in biotechnology and petrochemistry, rockets, robots, and automated systems. Thus, the change in the images of the Future also implied a visible change in their material carriers, which made it possible effectively to block only some directions of scientific and technological development by economic and political means, relying on the instruments of state regulation in public spheres. Suffice it to recall cutting the funding for many space exploration and exploration programs or a ban on developments in the field of genetic engineering.^2^

The emergence of an invisible digital code, a pure product of human thought or AI, has fundamentally changed the situation in the field of human interaction and digital technologies. Unlike other scientific and technical fields that a person encounters in the process of his socialization, now digital technologies, at least in developed countries, surround him from infancy. In fact, a modern person from birth finds himself in the digital world; therefore, for social and personal orientation in this habitat, a person is forced to acquire the appropriate skills and experience since his symbiosis with the digital reality will continue until the end of the century. As a result, a person’s understanding of the structure of the digital world, one might even say the digital Universe, his “perception of the digital world (for example, as given in comparison with something that is produced and that can thus be formed) determines what types of future are thought of as possible” [Dufva and Dufva, March 2019, p. 18].

Digitization is based on computer simulation based on *virtual reality*, often of a gaming nature. Virtual reality is based on imaginative, one might say artistic, thinking, as pointed out by decision-makers *using* computer programming. In particular, the Dutch analysts I. Cattenburch and M. Duijn, based on the experience of using digital technologies, came to the conclusion that artistic metaphors serve as mental models in the development and implementation of visual concepts. It is mental models that use images to understand clearly how things fit together, since metaphors are “ideal for isolating the main meaning (or meanings) when processing large amounts of data, forming a flexible framework for understanding and interpreting information” [Van Cattenburch and Duijn, March 2019, p. 108].

Image metaphors carry a moral component; for example, this is manifested in ancient Greek myths. In particular, the myth of Phaeton tells about the moral inferiority of the proud Phaeton: having taken the solar chariot from his father Helios for a short time, he lost control, and the horses carried him along the wrong trajectory to the planet Earth. As a result, he died, struck down by Zeus’s lightning. Metaphorical modeling of the Future with the help of digital technologies has one more important feature. It is related to programming the role that its creators intend to play in the Future. In essence, this is a scenario for managing and manipulating the structural and functional characteristics of the digital Future by modern political, financial, and economic elites, which begins with determining their focal location in the digital Universe. In this regard, the same ancient Greek myth about Phaeton gives an idea not only of the unlimited possibilities that the digital control of the world of the Future creates (Helios’s solar chariot), but also of the dangers that may arise for the ruling elites if they fail to cope with its management. In fact, the digital project of the Future involves the construction of a hierarchical social order, which has always been present in all socio-economic systems from ancient times to the present day. The blueprint for a digital future will invariably be the product of “elite visionaries” and “the dreams of the profane masses,” as most of the world’s masses “are in no position to anticipate for themselves either immediate benefits or improved long-term prospects from the forward march of technology. They must accept the promise of benevolent outsiders that their lives will be bettered through inventions designed elsewhere, by entrepreneurs closer to technology’s moving frontiers, with the capital and knowhow to engineer large-scale change. Inequality, not only as access but even more of anticipation, thus emerges as an unresolved ethical and political barrier to the just governance of technological innovation” [Sand, March 2019, p. 101].

## THE CORONAVIRUS PANDEMIC: IS IT A HARBINGER OF THE DIGITAL FUTURE?

The coronavirus pandemic that hit humanity at the beginning of 2020 has radically changed the usual rhythms in functioning of almost all public spheres and most states on planet Earth. The World Health Organization (WHO) declared the coronavirus outbreak a global epidemic on March 11, 2020 [6], and this date can be considered a conditional starting point for the approaching Future. As of spring 2022, 471.0 million people in the world had been infected with the coronavirus and almost 6.1 million people had died. It should be noted that the countries of the Americas and Europe account for 73% of all cases of coronavirus infection and just over 75% of COVID deaths [7].

Yale University history professor F. Snowden has studied the impact of pandemics on social development from 1346 to 1953, which claimed from 75 million to 200 million lives on the planet,^3^ starting from the time of the first world plague pandemic, and ending with the global pandemics of our time. He concluded that pandemics, like revolutions, wars, or economic crises, had invariably appeared to be turning points in the development of individual societies and of all humankind as a whole. The fundamental reason is that pandemics “reach into the deepest levels of the human psyche. They pose the ultimate questions about death, about mortality: What is life for? What is our relationship with God? If we have an all-powerful, omniscient, and benign force, how do we reconcile that force with these epidemics that sweep away children in extraordinary numbers?” [8].

As a rule, pandemics have led to a sharp increase in the role of the state and authoritarian forms of government, while such an increase does not stem from an understanding of what measures need to be taken, but from the exact opposite premise: the authorities “…not knowing what to do, this gave the impression that they did: They knew what they were doing, and they were taking decisive measures. And so, it was thought that these sorts of measures would possibly be effective, and would certainly be a display of power and resolution” [9].

The current situation, in particular, has already turned into a tectonic shift in the internal political situation of the United States. According to F. Snowden, the impact of the pandemic on the presidency of Donald Trump was fundamental. “Indeed, my view is that” writes the scientist, "without Covid-19, he would have been successful in his bid for re-election.” At the same time, as the American historian pointed out, D. Trump’s defeat goes beyond the traditional political theory of presidential elections and, perhaps, is important for representatives of the political elite in other countries. The pandemic has radically changed the usual tactics and strategy of conducting political campaigns, since for D. Trump, “he suddenly confronted an adversary that was a force of nature rather than a human rival. His normal strategy of bullying, and presenting his own ‘alternative facts’ proved ultimately of no avail when Covid-19 advanced remorselessly. In so doing, the pandemic exposed Trump’s incoherent response to the crisis, and it generated enormous suffering and death for which he was unwilling to accept responsibility. He also failed to convince the country that he possessed a solution to the greatest medical crisis of the century.”

The coronavirus pandemic has sharply increased the social control of state bodies over citizens based on digital technologies. At the same time, as F. Snowden emphasized, “What is new in the time of Covid-19 is that in some countries the authorities have deployed electronic monitoring devices in ways that George Orwell and Aldous Huxley would have understood. In those places, states have used drones and video cameras to exercise surveillance, have tracked the movements of individuals by employing their cell phones as tracking devices, and have used robots in health centers. Here there are new temptations for authorities to maintain the control, the surveillance, and the invasion of privacy after the emergency has passed. The boundary in these contexts between protecting health and promoting abuse of power is porous and ever shifting as the technology evolves” [10].

The pandemic has affected the mechanisms underlying the functioning of almost all public spheres. However, three main trends have already emerged in most countries that will determine the course of social processes in the near future. ***The*** ***first trend*** is associated with a noticeable increase in large technology firms actively developing and implementing digital technologies. ***The*** ***second*** comes down to an even greater increase in all types of inequality, especially socio-economic, which is stimulated by modern digital technologies, while it affects the distribution of economic benefits, political influence, and social relations, including gender, racial, age, and educational inequality. And, finally, ***the third trend*** manifests itself in the proliferation of information flows in the media, which are interpreted by certain segments of the audience as misinformation. Actually, it can be assumed that modern digital platforms have become objects of ***information wars*** going on both within societies and in the global cyberspace.

In general, these trends create “the new normal” situation in the future for the next five to ten years. [11]. At the same time, public life will be increasingly defined by concepts such as the “inflection point,” “punctuated equilibrium,” “inconceivable proportions,” an “exponential process,” “mass disruption,” and an “unprecedented challenge.” The rapidly evolving processes leading to the digitization of societies will unfold in conditions, as the famous American sociobiologist E. Wilson put it, of “Paleolithic emotions, medieval institutions, and godlike technology” [12].

Social distancing, which has become a legitimate form of combating the coronavirus pandemic, will gradually develop into a system of “tele-all,” that is, a system of remote healthcare, education, work, entertainment, e-commerce, and social events, including participation in political processes, which includes remote voting [Travkina and Rogovskii, 2016]. Social distancing in the broad sense of the word will mean that social communities, “individuals, cities, and nation-states will become more insular and competitive as survival mode kicks in. Xenophobia, bigotry, and closed communities will also increase” [11].

Social distancing has already led to serious psychological stresses. In the near future, we should expect an increase in psychopathic forms of behavior at the individual and group levels provoked by further complication. According to American analysts, problems and challenges, programs and technologies, everything will become more complex. “The substrate of the new normal will be ineradicable complexity: Both our problems and our technologies (including how we deploy these technologies) have passed the stage of simple approaches” [11].

As it is seen today, the complication of all social processes will create an increasing burden on the psyche, which threatens not only an increase in the number of mental disorders and diseases, but further change in the consciousness of mankind, which will take on such a wide scale that it can be put on a par with planetary climate changes [Davies, October 2016, p. 2139]. For example, the coronavirus pandemic has clearly revealed a trend towards a rise in mental illness and disorders and an absolute and relative increase in the number of suicides and the use of drugs and alcohol.

Thus, in the United States, according to American official statistics, in the first half of 2020, the proportion of the adult population reporting symptoms of an anxiety and/or depressive disorder increased almost fourfold compared to the first half of 2019, from 11.0% to 41.1%. At the same time, social groups with low incomes, representatives of ethnic minorities, and youth were in a particularly vulnerable position. In particular, in 2020, the proportion of the population aged 18 to 24 years who reported symptoms of anxiety and/or depressive disorder was 56.2% and the proportion of the population aged 25 to 49 years with similar symptoms was 48.9%. The share of the population aged 50 to 64 years with anxiety or depression was 39.1%; and the share of the elderly, only 29.3%, that is, almost two times less than the younger generation [13]. It is quite possible that the processes of further social digitization will be comparable in their medical and biological consequences to the impact of the 2020 coronavirus pandemic.

## “THE DIGITAL DIVIDE”: THE MAIN FACTOR OF EMERGING UTOPIAS AND DYSTOPIAS OF THE DIGITAL ERA

The distribution and implementation of digital technologies in public spheres is uneven and contradictory; as a result, a situation arises when some communities have access to and widely use the fruits of digitization while others are deprived of this opportunity. Inequality in available access to and ownership of digital technologies creates the phenomenon of the “digital divide.” It has many dimensions, but three of its types are considered the most referentially significant, i.e., (1) the gap between urban areas and rural settlements; (2) the gap between different kinds of socio-economic groups; and (3) the global gap between developed and developing countries. Thus, in 2020, approximately 4% of all households in the UK, i.e., over a million people did not have access to the Internet, even though the coverage of British households with the Internet proceeds “by leaps and bounds.” In 2000, only 25% of British households had access to the Internet, but by 2010 it was already 73% [15]. In addition, “the rural telecommunications infrastructure is inferior to that serving urban areas. This results in large numbers of people being unable to exploit fully the potential of ICTs because of where they live and work: yet there is a paucity of literature about the specific spatial nature of rural digital exclusion and the ramifications of this” [Philip, 2017, p. 387].

Socially, inequalities in educational attainment and income distribution in virtually every society play a critical role in shaping and deepening the digital divide. According to statistical surveys, people with higher and incomplete higher education have the potential to use digital technologies on average ten times higher than the same indicator for people with secondary and incomplete secondary education. High-income earners (individuals and households) ($75 000) are 20 times more likely to access the internet and digital technologies than low-income earners ($30 000) [14].

The digital divide is especially acute on a global scale: half of the world’s population (which is almost 4.0 billion people!) do not have access to the Internet, and in most of the least developed countries, no more than 20% have access to digital technologies, naturally not the most advanced [16].

The emergence of revolutionary technologies as digital technologies stamped themselves from the very beginning, could not but overlap with the structure of the eschatological consciousness of modern humanity, which, since the industrial revolution in Great Britain in the last third of the seventeenth century, has invariably considered the emergence of technology as an opportunity for humanity to gain the long-awaited “keys to earthly paradise,” having saved most of the population from want, and a growing abundance of material wealth will result, if not in the elimination, then at least in a significant metamorphosis of the various hypostases of Evil.

Digital technologies, which from the very beginning reflected and multiplied the Good, the Bad, and the Ugly, inevitably gave rise to the utopias of a “social paradise” and the dystopia of a “social hell.” As noted in this regard by the American philosopher A. Feenberg, who specializes in the philosophical problems of modern scientific and technological progress, “Contemporary utopias are presented as breathless frontline reports on the latest R and D. These new utopias are inhabited by bioengineered superhumans networked in a universal mind or downloaded to more durable hardware than the human body. Big data will soon predict when we will catch a cold and finally make possible a true science of society. Networked artificial intelligences will serve all our needs and eliminate work” [Feenberg A., 2017, no. 20: 78]. The coronavirus pandemic could not fail to highlight the dystopia of the “digital hell” approaching humanity, at least for those countries and social groups that will not be able to adapt and master modern digital technologies.

## DYSTOPIA: “DIGITAL SLAVERY”

The rapid spread of digital technologies in the political sphere has provoked a lively debate about the fate and prospects of the liberal democractic system, not only in Western countries, especially in the United States, but throughout the world. Digital technologies create objective opportunities for controlling large social groups of the population and individuals, as well as for strengthening possible repressive measures against them. In this regard, it is extremely significant that famous F. Fukuyama who proclaimed the global triumph of the ideas of liberal democracy directly linked the decline of democratic institutions in the leading Western countries, and especially in the United States, with the spread of digital technologies, which in the last decade contributed to a significant fragmentation of society and the declining confidence in state institutions.

As F. Fukuyama pointed out, even in the most democratic societies, “the emerging "internet of things” is gathering mind-boggling mountains of information whose uses will be even more opaque to individual users than is the case with today’s internet. Large and technically adept organizations, whether governments or private companies, can exploit “big data,” however, and are already beginning to do so. None of this is likely to bode well for democratic empowerment, though we are way too early in these developments to predict their political consequences" [Fukuyama, January 2020, p. 16].

Nevertheless, modern digital technologies at the disposal of political elites open up enormous opportunities for manipulating the moods, expectations, and value orientation of the broad masses of the population with political rights, which in ancient Greece were called “demos.” Modern ICTs make it possible to create a powerful system of imputed political values and ideas that reflect the ideas of political elites about the degree of independence for the “demos” in developing their own political views. Thus, only narrow groups of political elites remain the main political players in the pseudo-democratic political system, which, in relation to the “demos,” begin to pursue a policy of leveling it, which the German political scientist L. Ulbricht figuratively called “demos scraping.” According to her expert opinion, “in the guise of digitally enhanced democratization, a turn towards technocratic take-over and depoliticization is happening. Demos scraping, in its present form, is a Trojan horse for technocratic surveillance capitalism and an aesthetically pleasing materialization of simulative democracy” [Ulbricht, 2020, no. 3, p. 438].^4^

The loss of political rights opens the way for the next stage of “digital enslavement,” the loss of socio-economic rights and well-being: free citizens are gradually turning into “digital slaves.”

## UTOPIA: “DIGITAL PARADISE”

The visions of a “digital Hell” in today’s world are countered by the slickly beautiful pictures of a “digital Paradise.” An example of this kind of scenario is the analytical work “Digital Europe” prepared by a group of European researchers for the European Commission and published in the spring of 2019. This document contains a list of fundamental principles, the implementation of which will create “a new, society-centric vision that is intended to guide policymakers and civil society organizations in the direction of a more equitable and democratic digital environment, where basic liberties and rights are protected, where strong public institutions function in the public interest, and where people have a say in how their digital environment functions.” The writers of this script firmly believe that “Europe has every opportunity to create this kind of digital society” [20, p. 5].

The concept of creating a pan-European “digital Paradise” proceeds from the fact that at present in European countries there is a lack of digital technologies and, in general, digitization has not revealed its potential and its capabilities to the full. Actually, the authors of this scenario believe that all the problems of European digitization are due to the lack rather than excess of digital technologies. Creation of a “digital Paradise” should be based on four fundamental principles.

According to ***the*** ***first principle***, personal self-determination should be enabled, that is, opportunities for full participation in social life, including remotely, without the need to transfer personal data to commercial organizations, should be expanded. Self-determination includes the right to privacy and participation in more democratic models of data governance and algorithmic transparency.

According to ***the*** ***second principle***, a system for cultivating the commons should be developed. This principle assumes that Europeans, through digital technologies, should participate in joint work activities and exchange relevant knowledge for this purpose. Joint labor activity will be of great social value for all Europeans.

According to ***the third principle***, a consistent policy of decentralization of the European technological infrastructure should be pursued, which will allow them in the future to increase the technological sovereignty by reducing the dependence on non-European technology suppliers. Technological sovereignty is also a form of strengthening European democratic traditions and historically established cultural diversity in Europe.

Finally, according to ***the fourth principle***, state government bodies should be empowered, which will ensure the broad participation of citizens of European countries in the management of the education system, science, and culture. State institutions must be strong and effective enough to be able to provide online social services to the public, which must be reliably protected from the control of commercial Internet platforms [20, pp. 14–23].

Thus, while dystopias suggest an implementation mechanism based on the principle of self-fulfilling prophecies, the implementation of utopias comes from project plans implemented by powerful state institutions and global ICT corporations such as Apple, Microsoft, and Facebook. The Digital Future is a man-made project, mistakes and miscalculations in the development of which can have serious and even catastrophic consequences for the further historical evolution of individual countries and all of humanity, and which, apparently, can no longer be corrected by the “virtuous” hand of invisible Providence.

## CONCLUSIONS

### The Digital Future: Back to the Past?

The colossal increase in the economic power and political influence of global ICT corporations during the global pandemic crisis in 2020–2022 prompted many US political scientists, sociologists, economists, and state scientists to turn their attention to the American experience of the 1930s, to the New Deal policy of F.D. Roosevelt, who not only managed to use all the potential power of the state apparatus at the federal level to curb the undivided domination of the largest US monopolies of that period but also formed a new “social contract” of American society, which provided it with 30 post-war years of sustainable economic development. Study and reference to the experience of the “New Deal” is increasingly leading American social scientists to the idea that the time has come, at least in developed countries, especially in the United States, to develop and begin implementing the “Digital New Deal,” which would essentially allow for a “digital democratic revolution,” which put the digital sphere under the effective control of the masses [21, p. 9].

## Footnotes

1 And man is “led by an *invisible hand* to promote an end which was no part of his intention. Nor is it always the worse for society that it was no part of it. By pursuing his own interest, he frequently promotes that of the society more effectually than when he really intends to promote it” [Smith, 2008, p. 332].

2 In particular, according to a survey by the UNESCO International Committee on Bioethics, in 29 out of 39 countries actively involved in genetic engineering, there are bans on editing the human genome (that is, obtaining a genetically modified embryo), while in 25 countries such experiments are legally prohibited [5, p. 88].

3 In 2019, F. Snowden published a monograph on the impact of global pandemics on social development [Snowden, 2019].

4 American political life in the past decade provides excellent examples of the gradual transformation of a significant part of the American voters into a politically manipulated “demos.” Thus, in 2012, Republican presidential candidate M. Romney described 47% of American voters voting for the Democratic Party as “social dependents” [17]; in 2016, Democratic presidential candidate H. Clinton scorned D. Trump’s supporters as “a bunch of deplorable people” [18]; and, finally, US President J. Biden in March 2021 described tens of millions of D. Trump supporters as “having Neanderthal thinking” [19].
